# A questionnaire for a conceptual framework and interdisciplinary public health research using the Delphi technique—development and validation

**DOI:** 10.3389/fpubh.2025.1436569

**Published:** 2025-04-01

**Authors:** Anita Alaze, Emily Finne, Oliver Razum, Céline Miani

**Affiliations:** Department of Epidemiology and International Public Health, School of Public Health, Bielefeld University, Bielefeld, Germany

**Keywords:** questionnaire development, Delphi survey, interdisciplinarity, public health, conceptual framework

## Abstract

**Background:**

The Delphi technique has become established in public health research, yet there is a lack of methodological standards in questionnaire development. We here demonstrate how the Delphi technique can be used in an interdisciplinary public health topic for framework development, and we highlight methodological challenges and possible solutions.

**Methods:**

We developed the questionnaire through a comprehensive literature review and the generation of an item pool based on the rules of item construction. We used cognitive interviews, a Delphi experts assessment and group discussions to refine the questionnaire and to ensure content validity. Finally, we carried out a pre-test of the online questionnaire.

**Results:**

The questionnaire consists of three main sections, namely gender (norms), the social environment and the mental health of adolescents, and another section on characteristics of the panelists. It comprises a total of 32 questions and includes rating and ranking questions, content-related and comment questions, open and closed questions as well as questions on personal characteristics and evaluation questions.

**Conclusion:**

To address challenges in the development process, interdisciplinary researchers need to be involved. They should consider certain aspects of the development process to provide more structure and clarity, such as comprehensively preprocessing the content, and disentangling and simplifying the theoretical concepts. They should consider a rigorous approach to develop more complex frameworks for interdisciplinary public health topics. Future research should focus on developing methodological guidelines and testing their applicability for different objectives of the Delphi technique (e.g., framework development).

## 1 Introduction

The *Delphi technique*, an anonymous expert-based assessment process carried out in a group communication process, is used by researchers if the available knowledge on a topic is uncertain or incomplete. The procedure is as follows. The topic under investigation is evaluated by experts in an iterative process (1). A Delphi study consists of several (2–4) rounds until a consensus is reached, ideas are aggregated, future predictions are made or experts’ opinions are determined (2). After each round, the answers are summarized and attached to each new questionnaire so that the panelists surveyed can reconsider their opinions and revise them if necessary (2). The Delphi survey, compared to traditional group discussion, has numerous advantages. For example, flexibility, limiting dominance of certain individuals, limiting moderator biases, enabling collection of opinions from participants located in various geographic regions and at the same time ensuring anonymity during the survey (3). The objective for using the Delphi technique differs between disciplines. In public health, it is commonly used to find expert consensus. While the Delphi technique has gained acceptance in diverse fields of medicine and public health, little methodological guidance and quality standards are provided in the context of theory development (4). Thus, Delphi can be considered a technique rather than a fixed method. This leads to a lack of clarity on best practice in the application of the Delphi technique for framework development and even more so with an interdisciplinary and international Delphi panel. A systematic review revealed that only one out of 80 included studies provided the Delphi questionnaires in the appendix (5). It also shows that many essential elements of the Delphi technique are not reported in the studies (5), which indicates the need for improving the use and the reporting of the Delphi technique.

Adolescents experience sex differences in morbidity and mortality. Key determinants to explain these differences are socially constructed *gender norms* (6). These gender norms shape the way adolescents interact, form relationships, and engage in sexual and reproductive practices and social behaviors (6, 7). However, little is known about the factors that influence young adolescents’ gender norms and behaviors in relation to (mental) health (6). Gender theory highlights the importance of a multidimensional *social environment* shaping the various gender norms and attitudes of individuals. Structural dimensions, such as institutional laws and policies as well as social structure and individual aspects are often mentioned as influential factors in the macro, meso-and micro-environment associated with health (8–11). Adolescents’ social environment determines how adolescents learn about and construct gendered attitudes and norms which in turn affect health (12). This is why shaping these norms is particularly important. Differences in *mental health* status can be observed very early in life. Childhood and adolescence are critical periods of health promotion, as more than half of mental health disorders start at these stages and continue into adulthood (13). Rates of common mental health problems, such as depression or anxiety rise sharply in adolescence and peak in young adulthood (14). Depression, anxiety and behavioral disorders are among the leading causes of illness and disability, and suicide is the fourth leading cause of death among adolescents (15). Despite the multidimensional nature of this global challenge, many interventions persist in seeking solutions that only tackle the individual level (13).

*This study* aims at demonstrating how the Delphi technique can be used in an interdisciplinary public health topic for framework development. It can be used in quantitative research, and contributes to providing a foundation for more theory-driven research. Moreover, this article highlights methodological challenges and what has proven successful in framework development with an interdisciplinary Delphi panel. Furthermore, we will derive practical recommendations for other studies. We will illustrate this using the example of the development of a questionnaire for a Delphi study. The objective of the questionnaire is to analyze how (a) gender norms, (b) the social environment, and (c) the mental health of adolescents are theoretically connected and which are the most important associations between the identified constructs. In the following, we refer to gender, the social environment and the mental health of adolescents as the three research topics. We understand constructs as the key components of the questionnaire, such as mental health outcomes, and a range of gender concepts and approaches, among others. We include gender norms, sex and gender identity, for instance, under the umbrella term of gender concepts and the intersectionality approach and the multidimensionality approach, for example, under the umbrella term of gender approaches. We use the term item to refer to a questionnaire question with the corresponding answer option. There is clearly a need for a conceptual framework to map the interplay of the three topics under investigation. However, so far there is no such theoretical approach and also none that applies it to the age group of adolescents.

## 2 Materials and methods

### 2.1 Questionnaire development and validation process

The selection criteria for the panelists in the Delphi study are (i) researchers or work in development, services, or implementation (e.g., Non-Governmental Organizations, Civil Society Organizations, (inter-)governmental agencies), (ii) have sufficient written English and computer skills, and (iii) work or have worked on at least one of the three research topics (e.g., gender, gender socialization, mental health, adolescence, or multiple combinations of those). We further decided to include panelists from the Global North and South due to the global character of the research topic. We also paid attention to include gender non-binary panelists and panelists of different ages to obtain a sample with diverse perspectives on the research topics. We restricted the Delphi study to three rounds *a priori* due to the time-consuming nature of the Delphi technique and the complex topic. These methodological decisions for our Delphi study influenced the design of the first questionnaire. For example, the questionnaire must anticipate and address a variety of language, cultural and knowledge differences. Only few panelists have specialized knowledge in multiple scientific fields. This is why we disentangled and simplified the content to make the questionnaire understandable for panelists without specialized knowledge in these fields.

**Figure 1:**
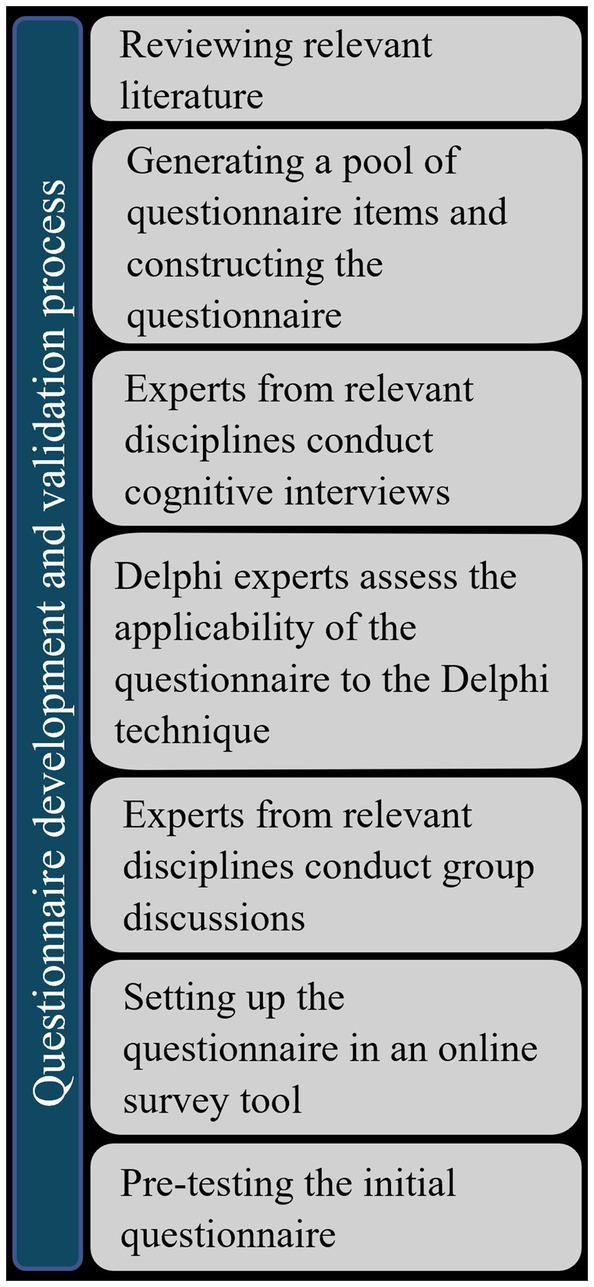
Development and validation process of the Delphi questionnaire.

The questionnaire development and validation process (see Figure 1) comprised (a) a comprehensive literature review to identify the content domain and theories, models or conceptual/theoretical frameworks within the three research topics, (b) the generation of a pool of instrument items based on the literature review and on the first author’s expertise and (c) the first set-up of the questionnaire. We generated all items according to the rules of item construction and for their use in quantitative research (comprehensive, positive, short, easy to understand, uniqueness, no universal expressions, no suggestive questions, no redundancy, closed questions to increase economy and objectivity of analysis) (16). In addition, we adapted the items to typical response scales of Delphi surveys, such as rating and ranking scales. After the first set-up of the questionnaire, we held (d) several cognitive interviews with one content expert per research topic to refine the questionnaire and to ensure content validity (17). (e) Two Delphi experts then evaluated the questionnaire for its appropriateness for a Delphi survey based on essential elements of the Delphi technique (e.g., length of questionnaire, type and formulation of questions and answer options, fit of study objective with selection criteria (validity, feasibility, importance, agreement or reliability), overall outlay of the Delphi study, and definition of consensus). We chose a maximum of three Delphi rounds because of the time load for the panelists and established a 70% threshold for consensus. This requires that 70% of the panelists rate a questionnaire item as at least fairly important. We selected this consensus threshold because it lies between the simple majority of the sample (51% agreement) and a rigorous threshold of 90%. We assumed that the interdisciplinary nature of the topic, with panelists with differing fields of expertise, could lead to a wide range of responses.

We implemented the resulting suggestions for improvement, item prioritization and reduction through group discussions with two interdisciplinary experts. We conducted (f) another cognitive interview with one psychology practitioner to assess the feasibility of the questionnaire for practitioners. (g) We implemented all findings in the questionnaire and (h) set them up on LimeSurvey, an online survey tool hosted on a server at Bielefeld University. (i) Ultimately, we pre-tested the questionnaire on LimeSurvey.

### 2.2 Literature review

We conducted a non-systematic search for theoretical frameworks on Medline (via PubMed) and websites of international organizations, such as UNICEF, WHO, and the UN. Moreover, we applied the snowball principle to search for more literature. We included theories, models or theoretical frameworks that covered at least one of the three research topics. They needed to be related to a health problem and should be used in public health research, health policy and implementation or gender studies. A requirement for including frameworks or theories was their applicability to public health research. We assessed this by identifying topics or factors that are integrated into public health research or practice, such as the social determinants of health or other common theories in public health. Our objective in selecting theories, concepts or frameworks was to inform the state of the art of the three topics - either to better understand key aspects of the specific topic or to find connections between them. This resulted in the generation of an item pool and the first draft of the questionnaire.

### 2.3 Expert interviews for content-validation of the questionnaire

#### 2.3.1 Cognitive interviews

We carried out cognitive interviews with three experts with expertise in at least one of the research topics. This was done to ensure that each topic in the questionnaire covers the relevant content. During the interviews, the experts assessed the understandability of the questions for the panelists, also in the areas where the panelists may not have expertise. This is an important step to address the interdisciplinarity of the questionnaire during the development phase.

We held the first round of cognitive interviews after the first set-up of the questionnaire items. Then, we conducted the second round of cognitive interviews with a child and youth psychologist after the group discussion (step 6) to assess the understandability of the questionnaire by practitioners. The procedure was as follows: The experts received the questionnaire by email to think about the questionnaire in advance. During the interview (mostly via Zoom), we reviewed the questionnaire from top to bottom and asked the experts to comment on several aspects: the understandability, completeness and relevance of the selected questionnaire items, accompanying response formats and missing or redundant items (content validity). We directed emphasis on the part of the questionnaire where the expert had their expertise.

#### 2.3.2 Delphi experts

We contacted two Delphi experts via email who assessed if the proposed questionnaire is applicable to and appropriate for the Delphi technique and if the research objective can be reached. We chose these two experts based on their previous experience in carrying out Delphi studies and their methodological expertise with the Delphi technique. Ultimately, we adapted the questionnaire based on the expert’s comments, feedback and suggestions to fit the Delphi technique, which we achieved through a group discussion. Notwithstanding, there is no set standard for the Delphi technique, making it difficult to know what exactly is required for a questionnaire to be suitable for the Delphi method.

#### 2.3.3 Group discussion

We held group discussions to reduce the number of questionnaire items and answer options, to increase the clarity, to reduce the cognitive load, and to adapt the layout and design to make it more appealing. We chose two scientists with a background in sociology and psychology who are currently working as public health researchers. This allowed us to address the interdisciplinarity of and the intersections between the research topics. We sent the questionnaire to the two experts before the group discussions. Ultimately, the group discussions included two meetings which we held in person. A challenge during the discussions was to decide which aspects should be predetermined (e.g., the most essential dimensions or definitions) to shorten the length of the questionnaire and which should be left as response options.

### 2.4 Pretest of the questionnaire

We pretested the questionnaire with researchers from the School of Public Health at Bielefeld University specializing in Psychology, Health Economics, and Gender Epidemiology. They checked for spelling mistakes, layout, practicability, introductory text, ambiguities in questionnaire items, and the set-up in LimeSurvey. The input of these researchers in the pre-test did not shape the content of the final questionnaire but rather its set-up and layout in LimeSurvey. Since it is challenging to convince busy experts to participate in a Delphi study with several rounds, we decided to carry out the pre-test within the faculty to maintain the chance of a higher participation rate in the actual survey.

### 2.5 Ethical considerations

The Delphi study involving human participants was reviewed and approved by the ethics board at Bielefeld University, Germany, in May 2023 (application number: 2023–111 of 2023/05/22). We did not obtain written informed consent specifically for the pre-test procedures. This was not required as the experts did not provide any personal data.

## 3 Results

### 3.1 Literature review

We retrieved four relevant frameworks that consider gender constructs and seven frameworks that consider the topics gender and adolescence (see Table 1).

**Table 1:** Results of the literature review: frameworks considering gender constructs and the topic of gender and adolescence.

Gender construct	Gender and adolescence
INGER (Integrating Gender into Environmental health Research) multidimensional sex/gender concept with an intersectional perspective (11)	Ecological model for an enabling environment for shaping adolescent sexual and reproductive health (28)
The gender analysis framework (9)	GAGE’s conceptual Framework (29)
Dynamic framework for social change (10)	Multi-level framework of influence impacting gender socialization processes during adolescence (30)
Conceptual framework for structural elements of gender power relations that drive gender inequality (31)	Conceptual framework of social and gender norms and power for ASRH (Adolescent Sexual and Reproductive Health) (32)
	Integrated model of menstrual experience (33)
	Conceptual measurement framework for impacts of gender inequality on the well-being of children and adolescents (34)
	Conceptual framework for healthy adolescent sexuality development and its potential link with sexual well-being (8)

We found that the general objectives of the retrieved frameworks were to inform research, develop interventions for practice or theoretically frame a research project concerning gender norms and adolescence. The literature review revealed that the interplay of these three topics is not or only partly covered in the existing literature. No framework, concept or model addressed the specific conceptualization of gender norms and even less so for adolescents. Moreover, no framework addressed specifically gender and mental health. Mental health topics were rarely mentioned in conjunction with the other two topics, and when they were, it was only broadly acknowledged. We also found that the frameworks do not explicitly show what specific relationships exist between constructs. The combinations of topics that we found were, for example, gender and intersectionality, but unrelated to adolescents, mental health, and their social environment. Another example were gender-related aspects, such as menstruation, body image, or sexual and reproductive health, but unrelated to gender norms, mental health, or to other gender elements. The social environment leading to the integration of the multi-level approach plays an important role in the frameworks, however, the specific inter-relational factors between the environmental levels and gender norms and the adolescent’s internalization process were not illustrated. Consequently, there is still a need to justify and clarify the use of these theoretical groundings to advance gender theoretical development (18).

The conceptual framework to be developed in the Delphi study will fill some gaps in the existing literature. Three research topics will be combined: (a) different gender-related aspects including gender norms, (b) the social environment, and (c) adolescent mental health. The framework will show in which way constructs are associated, thus taking a first step toward disentangling the gendered pathways of mental health in adolescents. It will also integrate diverse lenses or perspectives found relevant in the literature, such as intersectionality, embodiment, or the power relations lens. These lenses derive from the state of the art and ‘gold standards’ in gender research. For instance, Hammarström and Hensing identified six gender constructs as central to health sciences: sex, gender, intersectionality, embodiment, gender equity and gender equality (19). We thus included aspects retrieved from those frameworks as questionnaire items to adhere to these standards.

### 3.2 Cognitive interviews, Delphi experts, and group discussions

The cognitive interviews revealed which items of the questionnaire were incomprehensible. In addition, they indicated the necessity to include additional answer options. We restructured some sections to gain more clarity.

The evaluation of the questionnaire by the Delphi experts about its applicability to the Delphi technique revealed that the questionnaire should be further shortened to approximately 8 pages. Moreover, the cognitive demand of the questions should be further reduced, just like the number of possible answers and open questions (e.g., one comment question per question and not per answer option). Additionally, the experts advised to reduce the amount of explanation texts since they might not be read. Thus, the questions must contain all relevant information to answer the questions.

The qualitative group discussions further developed the questionnaire items toward more clarity, to reduce the cognitive load, and to adapt the design/layout aspects. This was done to make the questionnaire more appealing and to ultimately improve its applicability for the Delphi study. An obstacle toward greater clarity and a lower cognitive load was the theoretical complexity. We included many different theories from different disciplines that contain very similar concepts but different names. We discussed how they can be combined into meaningful and distinguishable concepts. Therefore, we disentangled different gender terms and further reduced the number of answer options and comment sections (per section instead of per question). Further, we implemented text boxes for definitions of terms. Where necessary, we have also adapted definitions to reflect the disentangled concepts and revised them to the most up-to-date understanding. Moreover, we introduced rating and/or hierarchical questions instead of asking to select the three most relevant aspects as we had done before.

### 3.3 Pretest of the questionnaire

The pretest revealed ambiguities in the questionnaire items, and the need for a better structured introductory text so that necessary information about the survey can be seen without reading the entire text. Technical aspects of the online survey were adapted as well.

### 3.4 Questionnaire

The final questionnaire consists of three main sections and a fourth section about the personal characteristics of the participants. Questionnaire items include content-related closed questions as well as open comment questions and open questions. The sections are briefly presented next. The first section on gender (9 questions) introduces definitions of the key concepts which are sex, gender, and gender norms. The definitions are intended to ensure that the framework is based on a shared understanding that challenges a binary understanding of sex. Moreover, we ask the panelists to assess which aspects of the gender self-concept (identity) at the individual level are important. We propose various gender concepts and approaches to be incorporated into the framework, which we ask the panelists to rate by importance. Further, we ask which gender norms particularly influence adolescents and their mental health (Table 2).

**Table 2:** Overview of the constructs and items in the Delphi questionnaire.

Questionnaire sections	Constructs	Items
Gender	Gender concepts	Current sex/gender identity Sex/gender roles Sex/gender expressions Sex/gender relations Sexuality (Sexual orientation)
	Gender approaches	Gender continuum Multidimensionality approach Multi-level approach Intersectionality approach Gender power relations Embodiment approach Decolonial lens
	Gender norms	General gender norms Gender norms linked to mental health
Mental health	Outcomes for adolescent mental health potentially affected by gender norms	Mental, social and physical well-being Depressiveness Connectedness Body image Self-efficacy Self-esteem Coping Self-control Sense of coherence Happiness Life purpose Self-harm Suicidal behavior Resilience Substance misuse Risky behavior
Social environment	Social environment levels important for adolescents’ gender norms	Individual level Interrelational level Community level National level Global level
	Social environment actors that shape the gender norms of adolescents	School environment (teachers, classmates etc.) Media (e.g., television, newspapers, movies) Social media (e.g., Tik-tok, Instagram) Peers/friends Family Sport groups Faith groups Healthcare providers Political parties Law enforcement Civil society Non-Profit-Organizations
	Individual competencies of adolescents to navigate gender norms	Coping skills Agency Navigation Interpersonal relationship skills Critical reflection skills Mental health literacy Respect and empathy for others

The section on the mental health of adolescents (4 questions) focuses on which mental health outcomes best capture the impact of gender norms on adolescent mental health. We decided to exclude mental health disorders, as they were not the aim of our research. Therefore, we ask the panelists to rate several mental health outcomes by the level of importance, and to state if they can be linked to gender norms.

In the social environment section (9 questions), we propose five different social environment levels in an illustration. In addition, we introduce actors that may play a role in the adolescents’ gender socialization process. These actors should then be assigned to the proposed social environment levels. Furthermore, we provide various competencies that adolescents may need to navigate in their social environment. They should also be rated by the level of importance.

The last section deals with the panelists’ characteristics (10 questions). These encompass the year of birth, the country in which the panelist had the most working experience, the country in which the panelist currently lives and in which professional environment they are primarily situated (research or policy etc.). Furthermore, we ask the panelists to rate their expertise in the different research topics to gain a better understanding about their competences and their response behaviors in the questionnaires. In addition, we assess their sex assigned at birth, their current sex/gender identity, and their sexual orientation. Ultimately, we ask the panelists to evaluate the questionnaire. An additional document file shows the complete questionnaire in Supplementary material 1.

## 4 Discussion

We sought to demonstrate how the Delphi technique can be used in an interdisciplinary public health topic for framework development. We illustrate this with the development of an initial questionnaire on gender norms, the social environment, and the mental health of adolescents for the first Delphi round. The questionnaire development is often seen as the biggest challenge when using the Delphi technique (20). Frameworks developed in Delphi studies are typically less complex, summarizing all important aspects of the topic under investigation in the form of a list. They do not link different influencing factors nor do they incorporate theoretical aspects [e.g., (21–23)]. Our Delphi study could therefore serve as an example of how the Delphi technique can be used to develop more complex theoretical frameworks.

To ensure that the questionnaire meets methodological standards and adheres to guidelines (17, 24, 16), we developed the questionnaire through a literature review, and we tested for content validity through cognitive interviews, a Delphi expert assessment and group discussions. The validity checks ensure that the questionnaire is more likely to generate more targeted responses. So far, questionnaires for Delphi surveys are typically only tested with a standard pretest like that of the respective online tools (20). Furthermore, we involved international experts from different disciplines in the development process of the questionnaire to reflect these characteristics at this early stage.

We encountered several challenges during the development and validation process. They all stem from the characteristics of the resulting framework. The framework must conceptually link gender (norms), the social environment and the mental health of adolescents. For that, it needs suitable social environment levels that include relevant actors or stakeholders who are carriers of gender norms for or contribute to gender socialization in adolescents. In addition, it must conceptualize gender and provide a categorization of gender norms that are relevant to adolescents. Moreover, it needs mental health outcomes that are related to gender and gender norms so that effects and influences can be measured.

The first challenge was the interdisciplinarity of the questionnaire. Our goal was to develop a consistent framework for the three research topics that integrates different gender concepts and approaches. The panelists have different perspectives and priorities and operate with different definitions for terms. Thus, we adapted relevant definitions so that each panelist can follow the logic of the questionnaire items. Moreover, the gender concepts and approaches must be presented in a way that leads to meaningful answer options. The questionnaire items may need to ask for an aspect in a slightly different way or different answer options may need to be provided than in the retrieved frameworks from the literature review. One major decision was that the panelists should complete the entire questionnaire and not just the section that relates to their area of expertise. As a result, the panelists have to answer questions without special knowledge in these fields. However, this ensures that the intersections between the topics are covered. A cross-tabulation of the answers in higher and lower competence is intended to redress this issue. Questions on panelists’ self-assessed expertise are also missing in many Delphi surveys (20). Moreover, pop-up information boxes that contain definitions of specific terms are used to inform panelists without expertise in that field so that they can answer the question. It is important to emphasize in the questionnaire that the definitions of terms are only examples, so that panelists with high expertise will not fill in the answer differently simply because of a slightly different understanding of the provided definition. We used cognitive interviews and group discussions to ensure understandability for panelists from all included disciplines. Moreover, the introduction of comment boxes as the qualitative part of the questionnaire enables a more concrete understanding of the specific views and ratings of the panelists.

The second challenge was the cognitive load of the questionnaire. The objective of the questionnaire involves complex and theoretical questions. We needed to limit the cognitive load to address the generally high drop-out rate in Delphi surveys. To address this challenge, we introduced ranking and rating scales since their benefit lies in a relatively low cognitive and time load (25). This should help the panelists to reduce the number of possible answers to the most important and influential aspects. To avoid a “response set” where participants always tick the same answer, we also limited the amount of rating or ranking scales. Besides the introduction of those scales, it was necessary to set some assumptions to reduce the number of questionnaire items. For that, it was particularly useful to predetermine specific aspects, such as the definitions of essential terms, the inclusion of relevant aspects or a limitation to most relevant answer options. Pop-up information boxes also contribute to saving time for the panelists.

The third challenge was the quantitative operationalizability of the framework. This requires clear definitions of terms and concepts, and clear relationships between them. We therefore disentangled the different and often very similar parts of the theoretical concepts and provided answer options (and definitions) that are distinguishable from each other. Another measure was to preselect the most essential answer options and to constantly remind the panelists that we were only asking for the most relevant aspects of the topic under investigation. The exclusion of less important aspects can lead to panelists mentioning some of the aspects not listed. However, fewer less relevant aspects will likely be mentioned in this way.

We can draw the following lessons for future Delphi studies investigating interdisciplinary public health questions. First, it is important to comprehensively review the current evidence before developing the content of the questionnaire. The theoretical content needs to be disentangled and simplified so that researchers without specialized knowledge can participate in the entire Delphi study. This also enhances the clarity and consistency of the questionnaire. The design decisions of the Delphi study, along with the specific constructs required for the final framework, will shape the structure and development of the first questionnaire. Therefore, particular attention must be given to the questionnaire for the initial Delphi round, making the validation of its content crucial. Last, we highly recommend involving researchers from different disciplines in the development process to address interdisciplinary challenges at an early stage.

## 5 Limitations

An important limitation of this questionnaire development is that we did not statistically test the questionnaire on reliability and validity. This has several reasons. We did not develop a scale, a construct or a measurement instrument that quantifies well-defined concepts, as this was not our aim. Consequently, we could not test for construct validity because there are no interrelated constructs or one construct in its entirety in the questionnaire. We queried as many constructs and instruments as possible so that a conceptual framework can be developed.

Moreover, it is not common for Delphi studies to test their first questionnaire on reliability or validity (22, 23), and methodological guidelines do not require it (26). To ensure a rigorous approach, we tested for content validity in the questionnaire through cognitive interviews, group discussions, and a Delphi expert assessment. Also, the comment boxes in the questionnaire will allow the panelists to add other constructs once the Delphi study is carried out. When tools, models or frameworks have been developed through a Delphi study, it is state-of-the-art that they are validated after their development (27). This is why we plan to statistically validate the conceptual framework after its development with the Delphi study.

Another limitation is that we did not execute the literature review in the form of a scoping or systematic review. Furthermore, it remains possible that underlying assumptions about language and interpretations of statement wording are not shared between researchers and panelists. The closed questions may also restrict the depth of panelist response and thus the quality of data collected may be diminished or incomplete. We included panelists from both the Global North and South. This increases variability of perspectives and leads to more generalizable results.

## 6 Conclusion

Many Delphi studies do not report on essential elements of the Delphi technique, in particular the specific development steps and the exact questionnaire (5). We have addressed this gap by demonstrating how the Delphi technique can be used to develop a quantitative framework integrating three different public health-relevant topics and by providing the questionnaire of the first Delphi round. Future research should focus on developing methodological guidelines and testing their applicability for different objectives of the Delphi technique (e.g., framework development, interdisciplinary research). Our findings contribute to this endeavor by demonstrating the relevance of testing the content validity of the initial questionnaire, of simplifying complex content to create a clear and consistent questionnaire, of illustrating design decisions that shape the Delphi study, and of presenting a rigorous approach to develop better frameworks for interdisciplinary public health topics.

## Data Availability

The original contributions presented in the study are included in the article/Supplementary material, further inquiries can be directed to the corresponding author.
